# American Dental Association

**Published:** 1897-12

**Authors:** 


### Reports of Society Meetings.

### AMERICAN DENTAL ASSOCIATION.

(Continued from page 754.)

*August 4, 1897.—Second day.—Evening Session.*

The meeting was called to order at eight o'clock by the President, Dr. Truman.

The Secretary read the minutes of the previous session, which were approved.

Dr. Richard, President of the Southern Dental Association, was invited to a seat upon the platform next to President Truman.

Dr. Fillebrown read the report of the Committee on Union of the two societies, after which Dr. Marshall moved that the Committee on Union be requested to arrange for a convention of the members of the two Associations to consider the subject.

Motion carried.

Dr. Fillebrown, the Vice-President, then took the chair, and Dr. Patterson read the report of the Committee on the President's Address, as follows:

### REPORT OF THE COMMITTEE APPOINTED BY THE AMERICAN DENTAL ASSOCIATION TO CONSIDER THE RECOMMENDATIONS EMBODIED IN THE ANNUAL ADDRESS OF THE PRESIDENT, DR. JAMES TRUMAN.

To the Members of the American Dental Association:

Your committee confidently expect that the proposed union of the American and Southern Dental Associations will shortly be consummated, and their recommendations, therefore, are made to the Association about to be created by such union.

We recommend that all delegates to the Association shall be practitioners of dentistry, who shall be selected by State Dental Associations at a regular meeting of the association which they represent, and that they shall be men of recognized merit, and duly selected by a majority vote of such State organizations.

We recommend that one delegate shall be allowed for every ten members of such State organizations.

Your committee believes that with such careful election of delegates the formation of an aristocracy so forcibly described in your President's address would be prevented.

Your committee recommends the timely recommendation that some method shall be adopted by which a fund shall be created by the National Association which shall be devoted to original investigation, such fund to be used in enabling competent scientists in the profession to prosecute investigations without being harassed by the usual strife and annoyance encountered in dental practice.

Your committee is in perfect harmony with the President where he says that the degree of any State should be a law for all other States, and that a practitioner who is properly licensed in one State should be allowed registration in any, and that this Association should advise all of its members to work for the accomplishment of this desirable end.

In regard to the dental education and to the conflict which has arisen between the National Association of Dental Faculties and the National Association of Dental Examiners in respect to educational methods in the profession, to which extended reference is made in your President's address, your committee is of the opinion that there is no reason which should prevent harmonious relations between these two bodies. The aims of the National Board of Dental Examiners have been in the proper direction. They have been invested with authority by the various States to guard the community interests where they exist, and while individual members of the board have expressed unwise individual opinions, the public and majority action of the Examiners' Association has so far not exceeded their lawful and legitimate functions. They are entitled to our respect, and invite harmony with the National Association in dental education.

Your committee, while concurring in the majority action of the Examiners' Association, desires to pointedly criticise the method in some States of granting temporary licenses to practise, and would earnestly advise that efforts be made to change the laws permitting such licenses without any examination whatever.

In furthering the intent of the President's address, your committee desires to deplore the lack of interest in the national associations, evidenced by a considerable portion of both the membership of the National Association of Dental Faculties and the National Board of Dental Examiners, in that they have not and evidently do not desire to work with the National Association. So soon as the business of these two Associations is finished many of the members at once return to their homes. This is done in face of the fact that, of all associations, the National Board of Dental Examiners and the National Association of Dental Faculties should be the most loyal to national organizations, and thus further the community interests of dental education.

In conclusion, your committee feels that nothing will be gained by exalting the work of one or belittling the work of the other.

J. D. PATTERSON, *Chairman.*

G. MOLYNEAUX.

H. B. NOBLE.

Dr. Molyneaux then made some supplementary remarks regarding the President and his work in the profession.

Dr. Peirce moved that the report of the committee be accepted and taken up as the first order of business to-morrow morning.

The report of Section IV. was then read by Dr. Wilson; an abstract follows:

Section IV., Histology and Microscopy.

This section has sustained a great loss during the past year. Dr. Frank Abbott, who has been chairman of the section every year, with two exceptions, since the establishment of this branch of study in this Association, has passed away. On examining the transactions of this Association, I find that the first researches ever conducted in this country to determine the etiology and progress of the carious process in human teeth were conducted by Dr. Abbott in 1878; and I learn also, from the records, that nearly every pathological condition found in these important organs has been microscopically studied by him, and the results obtained have been from time to time reported to this Association. Only twice since the establishment of this section has he failed to contribute a valuable paper.

Dr. Abbott has kept this section alive. In 1894, at Old Point Comfort, he was the only member of the section present. But he realized, as we all must, that the wealth of this comparatively new field of research has not been exhausted, and has yet in store for us rich fruitage. Only a short time before his death he wrote me an earnest letter regarding the work of this section, and expressed a desire that we secure if possible a greater interest in this important line of investigation.

The Transactions of the American Dental Association for a score of years are richer because of his untiring researches. If the pages of our dental journals are to be regarded as an index to the histological and microscopical investigations of the past year, but few have been engaged in this line of study. It is with pleasure, however, that we call attention to a paper read before the New York Odontological Society last January by Dr. J. Leon Williams, of London, England, entitled "A Contribution to the Study of the Pathology of Enamel."

This paper, illustrated by about one hundred photomicrographs, was published in the *Dental Cosmos* of March, April, and May of the present year, and should be read and carefully studied by every member of the dental profession. This paper certainly marks an epoch in the development of dentistry. While it is true Dr. Black had in some sense foreshadowed what Dr. Williams has brought regarding the influence of films formed by micro-organisms, it has remained for Dr. Williams to demonstrate it. This clear demonstration by Dr. Williams fully merits the credit of discovery. His study of the teeth with reference to their liability to caries is also very important, especially the studies of the condition of the enamel.

One paper only has been secured for this section, by I. Norman Broomell, of Philadelphia, entitled "Macroscopic Tooth-Development," illustrated with lantern slides from original dissections.

*Dr. Crouse.*—I would like to have a committee appointed to audit the accounts of the Dental Protective Association.

*The President.*—I appoint as such committee Drs. Patterson, Rich, and Noble.

Dr. Fillebrown moved that this Association adjourn to-morrow at 12.30 o'clock, to meet with the Southern Dental Association in order to consider the question of union.

Dr. Broomell then read his paper, illustrated with various lantern slides. This very valuable contribution was mainly a description of this original work in the various pictures presented.

*Dr. Marshall.*—I want to express my appreciation of the exhibit upon which we have just looked. Any one who has ever attempted such dissections knows what an amount of work there is attached to it. Dr. Broomell is entitled to the thanks of this Association for what he has done, and I therefore move such a vote of thanks to him.

Carried unanimously.

Adjournment.

(To be continued.)

